# Maternal periconceptional folic acid supplementation reduced risks of non-syndromic oral clefts in offspring

**DOI:** 10.1038/s41598-021-91825-9

**Published:** 2021-06-10

**Authors:** Wenli Xu, Ling Yi, Changfei Deng, Ziling Zhao, Longrong Ran, Zhihong Ren, Shunxia Zhao, Tianjin Zhou, Gang Zhang, Hanmin Liu, Li Dai

**Affiliations:** 1 National Center for Birth Defects Monitoring, West China Second University Hospital, Sichuan University, No.17 Section 3 Renminnanlu, Chengdu, 610041 Sichuan China; 2 Sichuan Provincial Hospital for Women and Children, Affiliated Women and Children’s Hospital of Chengdu Medical College, Chengdu, Sichuan China; 3 Chengdu Women’s & Children’s Central Hospital, Chengdu, Sichuan China; 4 Pediatric Department, West China Second University Hospital, Sichuan University, No.17 Section 3 Renminnanlu, Chengdu, 610041 Sichuan China; 5 Key Laboratory of Birth Defects and Related Diseases of Women and Children (Sichuan University), Ministry of Education, Chengdu, Sichuan China; 6 Med-X Center for Informatics, Sichuan University, Chengdu, Sichuan China

**Keywords:** Diseases, Health care, Medical research, Risk factors

## Abstract

Maternal periconceptional folic acid supplementation (FAS) has been documented to be associated with decreased risk of nonsyndromic oral clefts (NsOC). However, the results remain inconclusive. In this population-based case–control study of 807 singletons affected by NsOC and 8070 healthy neonates who were born between October 2010 and September 2015 in Chengdu, China, we examined the association of maternal FAS with the risk of nonsyndromic cleft lip with or without cleft palate (NsCL/P), and cleft palate (NsCP). Unconditional logistic regression analysis was used to estimate the crude and adjusted odds ratios (ORs) and 95% confidential intervals (CI). Significant associations were found between maternal periconceptional FAS and decreased risk of NsCL/P (aOR = 0.41, 95% CI 0.33–0.51). This protective effect was also detected for NsCL (aOR = 0.42, 95% CI 0.30–0.58) and NsCLP (aOR = 0.41, 95% CI 0.31–0.54). Both maternal FAS started before and after the last menstrual period (LMP) were inversely associated with NsCL/P (before LMP, aOR = 0.43, 95% CI 0.33–0.56; after LMP, aOR = 0.41, 95% CI 0.33–0.51). The association between NsCP and maternal FAS initiating before LMP was also found (aOR = 0.52, 95% CI 0.30–0.90). The findings suggest that maternal periconceptional FAS can reduce the risk of each subtype of NsCL/P in offspring, while the potential effect on NsCP needs further investigations.

## Introduction

Oral clefts (OC), resulting from the incomplete fusion of primary palate and/or secondary palate during the 7th to 10th week of pregnancy, usually include syndromic or nonsyndromic cleft lip with or without cleft palate (CL/P) and cleft palate (CP)^1^. Depending on geographic regions and races, oral clefts affect 1 in 500 to 2500 births^1^. The nonsyndromic clefts (NsOC) are one of most common birth defects in China^2^. The reported prevalence of NsOC can be as high as 1.8/1000 in some provinces of China, causing huge disease burden on family and society^2–4^.

Maternal folic acid supplementation (FAS) before and during early pregnancy is a well-established prevention approach for neural tube defects (NTDs). As facial mesenchyme is derived from neural crest, the protective effect of maternal FAS on oral clefts has been studied in various populations, but the findings are inconclusive^5–8^. Most observational studies conducted in Europe^9–14^, America^15–17^, and Asia^18–21^, have shown that maternal FAS during early pregnancy is associated with decreased risk of nonsyndromic CL/P (NsCL/P), while other investigations in these areas did not^22–27^. Some of them revealed a preventive effect for nonsyndromic cleft palate (NsCP)^9,13–15^, whereas a few studies suggested maternal FAS or multivitamin intake as risk factor of NsCL/P and NsCP^28^. Though two recent meta-analyses support the potential preventive effect of maternal FAS against NsCL/P and NsCP^7,8^, the currently available evidence is insufficient and conflicting, particularly for some cleft subtypes^29,30^. In fact, population-based evidence from China is relatively scarce. With the exception of one cohort in three provinces^18^, a population-based case–control study in Shenyang city^21^, and a propensity-matched study in Shaanxi province^26^, almost all the observational studies in China have been hospital-based^31–35^.

Chengdu, the capital of Sichuan province, is a megacity located in the southwest of China where the prevalence of OCs is higher than the national average^3^. Following the Guide of National Folic Acid Supplementation Program^2^, Chengdu has provided free folic acid alone (400 μg/day) for women of childbearing age who reside in the area with a plan to be pregnant since 2010. The information regarding maternal folic use, prenatal exposures, perinatal health care and birth outcomes are prospectively collected by the Chengdu Maternal and Infant Health Surveillance system (CMIHS). These data allowed us to examine additional effect of maternal FAS on NsOC subtypes by performing a population-based case–control study.

## Results

### General characteristics of the study subjects

During October 2010 to September 2015, a total of 807 singletons with nonsyndromic clefts from CMIHS were available for the current analysis, including 247 NsCLs, 369 NsCLPs, and 191 NsCPs. Table 1 shows the maternal and infantile characteristics of the cases and 8070 controls. Compared with the control mothers, more NsCL/P mothers were under the age of 25 years or older than 35 years of age at the time of delivery. NsCL/P mothers were less educated (≤ 9 years), or more overweighted (BMI ≥ 24.0) than control mothers. Much more NsCL/P mothers exposed to environmental risks. Male predominance in NsCL/P and female excess in NsCP were identified. No significant difference was found between cases and controls regarding the distribution of parity, and maternal medical conditions in the first trimester.

**Table 1:** General characteristics of the study subjects.

Variables	Control	NsCL/P	NsCL	NsCLP	NsCP
	(n = 8070)	(n = 616)	(n = 247)	(n = 369)	(n = 191)
**Maternal age (years)**					
< 25	3058 (37.9%)	251 (40.8%)	95 (38.5%)	156 (42.3%)	65 (34.0%)
25–34	4495 (55.7%)	305 (49.5%)	130 (52.6%)	175 (47.4%)	111 (58.1%)
≥ 35	517 (6.4%)	60 (9.7%)	22 (8.9%)	38 (10.3%)	15 (7.9%)
**Maternal education (years)**					
≤ 9	2004 (24.9%)	172 (27.9%)	71 (28.7%)	101 (27.4%)	44 (23.0%)
10–13	2689 (33.3%)	226 (36.7%)	93 (37.7%)	133 (36.0%)	68 (35.6%)
≥ 13	3377 (41.8%)	218 (35.4%)	83 (33.6%)	135 (36.6%)	79 (41.4%)
**Urban–rural classification**					
Urban	2972 (36.8%)	180 (29.2%)	74 (30.0%)	106 (28.7%)	73 (38.2%)
Rural	5098 (63.2%)	436 (70.8%)	173 (70.0%)	263 (71.3%)	118 (61.8%)
**Residence registration**					
Local	7574 (93.9%)	577 (93.7%)	233 (94.3%)	344 (93.2%)	175 (91.6%)
Temporary	496 (6.1%)	39 (6.3%)	14 (5.7%)	25 (6.8%)	16 (8.4%)
**Maternal BMI (kg/m**^**2**^**)**					
< 18.5	1216 (15.1%)	81 (13.2%)	36 (14.6%)	45 (12.2%)	22 (11.5%)
18.5–23.9	5417 (67.1%)	392 (63.6%)	145 (58.7%)	247 (66.9%)	130 (68.1%)
≥ 24	1437 (17.8%)	143 (23.2%)	66 (26.7%)	77 (20.9%)	39 (20.4%)
**Parity**					
Primiparous	5342 (66.2%)	415 (67.4%)	155 (62.8%)	260 (70.5%)	124 (64.9%)
Multiparous	2728 (33.8%)	201 (32.6%)	92 (37.2%)	109 (29.5%)	67 (35.1%)
**Medical condition in the first trimester**					
No	7406 (91.8%)	562 (91.2%)	227 (91.9%)	335 (90.8%)	183 (95.8%)
Yes	664 (8.2%)	54 (8.8%)	20 (8.1%)	34 (9.2%)	8 (4.2%)
**Environmental exposure in the first trimester**^**a**^					
No	7901 (97.9%)	596 (96.8%)	243 (98.4%)	353 (95.7%)	186 (97.4%)
Yes	169 (2.1%)	20 (3.2%)	4 (1.6%)	16 (4.3%)	5 (2.6%)
**Family history**					
No	8068 (100.0%)	597 (96.9%)	242 (98.0%)	355 (96.2%)	188 (98.4%)
Yes	2 (0.0%)	19 (3.1%)	5 (2.0%)	14 (3.8%)	3 (1.6%)
**Infant sex**^#^					
Female	3919 (48.6%)	241 (39.1%)	96 (38.9%)	145 (39.3%)	109 (57.1%)
Male	4151 (51.4%)	374 (60.7%)	151 (61.1%)	223 (60.4%)	82 (42.9%)
**Gestational age (weeks)**^**b**^					
< 37	406 (5.0%)	401 (65.1%)	132 (53.4%)	269 (72.9%)	17 (8.9%)
≥ 37	7664 (95.0%)	215 (34.9%)	115 (46.6%)	100 (27.1%)	174 (91.1%)

NsCL/P, nonsyndromic cleft lip with or without cleft palate cases; NsCL, nonsyndromic cleft lip cases; NsCLP, nonsyndromic cleft lip with cleft palate cases; NsCP, nonsyndromic cleft palate cases.

^#^The infant sex records with one missing value on NsCLPs.

^a^The environmental exposure in the first trimester was whether the mothers had smoking, alcohol drinking, drug abuse, or exposure to radiation and hazardous substances in the first trimester.

^b^For those cases of termination of pregnancy, their gestational age at the time of termination of pregnancy were regarded as birth week.

### Maternal periconceptional FAS

As shown in Table 2, about 91% of control mothers and 81% case mothers had FAS during periconceptional period. More NsCL/Ps and NsCPs mothers did not take FA than control mothers (20.6%, 12.5% vs 9.2%). Among mothers who had FAS, 23.7% of control mothers, 21.3% of NsCL/Ps mothers, and 17.3% of NsCPs mothers initiated FAS before LMP. In addition, pregnant women greater than 35 years of age, with lower education level, or with BMI ≥ 24.0, were more likely not to have FAS.

**Table 2:** Maternal periconceptional folic acid supplementation in cases and controls.

Group	Control^a^	NsCL/P	NsCL	NsCLP	NsCP
	(n = 8070)	(n = 616)	(n = 247)	(n = 369)	(n = 191)
Non-users^b^	743 (9.2%)	127 (20.6%)	52 (21.0%)	75 (20.3%)	24 (12.5%)
Periconception users^c^	7327 (90.8%)	489 (79.4%)	195 (79.0%)	294 (79.7%)	167 (87.5%)
Preconception users^d^	1909 (23.7%)	131 (21.3%)	61 (24.7%)	70 (19.0%)	33 (17.3%)
Post-Conception users^e^	5418 (67.1%)	358 (58.1%)	134 (54.3%)	224 (60.7%)	134 (70.2%)

NsCL/P, nonsyndromic cleft lip with or without cleft palate cases; NsCL, nonsyndromic cleft lip cases; NsCLP, nonsyndromic cleft lip with cleft palate cases; NsCP, nonsyndromic cleft palate cases.

^a^Significant differences were found between controls, the overall and each subtype of nonsyndromic oral clefts.

^b^Non-users were defined as not having taken folic acid supplements or taken it continuously less than 1 month during the periconceptional period.

^c^Periconception users were defined as having taken folic acid supplements regularly at least 1 month during the periconceptional period.

^d^Preconception users were defined as having taken folic acid supplements regularly at least 1 month during the periconceptional period and started before their last menstrual period.

^e^Post-Conception users were defined as having taken folic acid supplements regularly at least 1 month during the periconceptional period and started on or after their last menstrual period.

### Association of maternal FAS and NsOC

As shown in Table 3, the crude odds ratio of maternal periconceptional FAS for NsCL/P was 0.39 (95% CI 0.32–0.48), and the adjusted OR was 0.41 (95% CI 0.33–0.51). Further stratification analysis showed that the associations remained significant for NsCL (aOR = 0.42, 95% CI 0.30–0.58) and NsCLP (aOR = 0.41, 95% CI 0.31–0.54). When analyzing according to the initiating time of maternal FAS, both maternal preconception and post-conception use could reduce the risks of NsCL/P and the subtypes (Table 3). Notably, maternal preconception FAS appeared to lower the risk of NsCP (aOR = 0.52, 95% CI 0.30–0.90). But the association between maternal periconceptional FAS and NsCP was nonsignificant. Several known risk factors such as advanced maternal age, higher maternal BMI, living in rural areas, exposure to environmental risks, nulliparity and male infants were positively associated with NsCL/P. Females were identified as risk factors of NsCP (Supplementary Table 1).

**Table 3:** The associations between maternal peri-conceptional folic acid supplementation and nonsyndromic orofacial clefts.

Cleft group^a^	Periconception users		Preconception users		Post-conception users	
	cOR (95% CI)	aOR^b^ (95% CI)	cOR (95% CI)	aOR (95% CI)	cOR (95% CI)	aOR (95% CI)
NsCL/P (n = 616)	**0.39 (0.32,0.48)**	**0.41 (0.33,0.51)**	**0.40 (0.31,0.52)**	**0.43 (0.33,0.56)**	**0.39 (0.31,0.48)**	**0.41 (0.33,0.51)**
NsCL (n = 247)	**0.38 (0.28,0.53)**	**0.42 (0.30,0.58)**	**0.46 (0.31,0.67)**	**0.51 (0.34,0.75)**	**0.35 (0.26,0.50)**	**0.39 (0.28,0.55)**
NsCLP (n = 369)	**0.40 (0.31,0.52)**	**0.41 (0.31,0.54)**	**0.36 (0.26,0.51)**	**0.38 (0.27,0.54)**	**0.41 (0.31,0.54)**	**0.42 (0.32,0.56)**
NsCP (n = 191)	0.71 (0.47,1.12)	0.69 (0.45,1.10)	**0.54 (0.32,0.92)**	**0.52 (0.30,0.90)**	0.77 (0.50,1.22)	0.75 (0.48,1.20)

NsCL/P, nonsyndromic cleft lip with or without cleft palate cases; NsCL, nonsyndromic cleft lip cases; NsCLP, nonsyndromic cleft lip with cleft palate cases; NsCP, nonsyndromic cleft palate cases. Bold values are statistically significant at α = 0.05.

^a^There were 8070 controls.

^b^Adjusted for maternal age, maternal education, urban–rural classification, residence, parity, medical condition and environmental exposure in the first trimester, maternal BMI, and infant sex.

## Discussion

In this population-based case–control study, we demonstrated that maternal periconceptional FAS was associated with a reduced risk of overall NsCL/P, and the reduced risk varied by cleft subtype and supplementation initiation timing. We did not identify the preventive effect of periconceptional FAS for NsCP, whereas we observed a significant association between maternal FAS started before LMP and NsCP, suggesting that earlier or longer supplementation may be protective.

Consistent with most previous studies in some western^9–17,36^ and Asian non-Chinese populations^19,20^, our results showed that maternal periconceptional FAS could reduce the risk of overall NsCL/P by approximately 60% regardless of the initiation timing. This preventive effect was observed for both NsCL and NsCLP. Similar associations have been noted in several epidemiological studies in China^18,30–34^^.^ These investigations were hospital-based and mainly focused on the environmental risk factors rather than the effect of supplementation. So far, only one cohort study specifically evaluated the preventive effect of maternal FAS on oral clefts in China, and found that in the north of China maternal supplementation started before the LMP reduced the risk of NsCL/P (NsCL/P, aRR = 0.69, 95% CI 0.55–0.87; NsCL, aRR = 0.26, 95% CI 0.07–0.98; NsCLP, aRR = 0.19, 95% CI 0.07–0.50), while such effect was neither observed in the south of China nor for supplementation started on or after the LMP^18,31–35^. Another population-based case–control study in Shaanxi province reported a preventive effect of optimal FAS for overall birth defects (OR = 0.71, 95% CI 0.57–0.89), but not for oral clefts^26^. The authors thought that differences in study design, supplementation initiation timing and small sample size could explain differences studies^18,26^. A recent meta-analysis revealed the preventive effect of maternal FAS in early pregnancy against NsCL/P and NsCP, and identified publication bias in previously published researches^7^. However, another systematic review seemed not to support the preventive effect of daily FAS on oral clefts^30^. It can be seen that inconsistences among various studies are obvious. In fact, there is still a lack of reliable evidence on the preventive effect of folic acid on oral clefts, especially on the dose and initiation timing of supplementation.

Nonsyndromic cleft palate has been regarded as a distinct condition from NsCL/P on embryologic origin and etiology. As noted in several previous investigation^6,11,18,21,31^, this study found a negative but nonsignificant association of maternal periconceptional FAS with NsCP. Interestingly, maternal FAS starting before LMP was significantly associated with decreased risk of NsCP, suggesting that earlier use may work or supplementation initiation timing may play a part. A few studies reported the preventive effect of FAS on NsCP^7,32^. Compared with NsCL/P, researches on FAS and NsCP produced more conflicting results. A cohort study in Norway reported an adjusted RR of 0.84 (95% CI 0.66–1.06) for maternal use of Vitamins/folic acid and NsCP, and a stronger association (RR = 0.63, 95% CI 0.45–0.88) for cleft palate in combination with other malformations^24^. On the contrary, a case–control study conducted in Northern Netherlands^27^ and another cohort study^28^ in Japan identified maternal FAS/multivitamin supplement use during the first trimester as a risk factor of NsCP. Whether the preventive effect of maternal FAS against NsCP can be observed in a certain population depends on various factors, including genetic background, maternal dietary folic acid intake, serum folate level and the compliance with FAS, etc. Considering the small number of NsCP cases in our analysis, the findings about maternal FAS and NsCP need to be further studied. These controversial results may be due to the heterogeneities in research design, population, sample size, exposure assessment, and other potential confounders^7,18,21,31–35^. Overall, more prospective studies are needed to elucidate the relationship between maternal FAS and NsCP.

Several strengths and limitations should be considered when interpreting our study results. The population-based nature and large sample size of Chinese cleft cases of the study ensure robust OR estimates for NsCL/P subtypes. In addition, the prospectively collected exposure data could minimize recall bias. When calculating the OR estimates for NsCP, there were potential bias due to low detection rate of syndromic CP but it could be minimal. Though information of maternal dietary and multivitamin intake was not available for analysis, the associations are less likely to be distorted because the OR estimates were based on randomly selected control data with adjustment for known risk factors such as maternal illness, social factors, environmental exposures, and maternal illness.

In conclusion, our study provides additional evidence that maternal FAS during periconceptional period can reduce the risk of NsCL/P. Larger-sample-size studies are warranted to elucidate the association with NsCP and to determine whether women can benefit more from supplementation starting before LMP. It is of paramount importance for women of childbearing age to become aware of that maternal FAS can not only reduce the risk of NTD, but also reduce the risk of NsCL/P.

## Methods

### Data source and study subjects

Data for this study were abstracted from the CMIHS. According to the “Maternal and Infant Health Care Protocol of Sichuan Province”^37^, every pregnant woman was required to have at least five prenatal medical examinations during her pregnancy and three postnatal clinical visits (on the 7th, 28th and 42nd day after delivery). The results of examinations or visits, risk factors (maternal diseases, family history, other medical conditions), and pregnancy outcomes (spontaneous abortion, stillbirth, live birth, birth defects, etc.), were recorded in the CMIHS system. The medical examination, data collecting, checking and auditing were performed by well-trained obstetricians and nurses. CMIHS adopted the diagnosis criterions, case ascertainment, quality assurance, data collection and encoding of birth defects proposed by Chinese Birth Defects Monitoring Network (CBDMN)^38^. In detail, nonsyndromic clefting referred a cleft case without any non-cleft malformations, while the syndromic cleft was defined as a case occurred in association with other congenital anomalies. The diagnosis was made by at least one obstetrician or pediatrician, and confirmed by a senior doctor at the hospital level. Following the CBDMN guide, the diagnosis was finally coded by CMIHS stuff according to International Classification of Diseases, Tenth Revision. Prenatally diagnosed cases must be rechecked and confirmed after birth. Between October 2010 and September 2015, 807 singletons with NsOCs were included in as the cases, for each case ten controls were randomly selected from the healthy singletons born in the same period.

### Folic acid intake and covariates

Briefly, women who joined in the prevention program were followed once a month by local community healthcare workers, and their registration dates, last menstrual period (LMP), dates of starting and ending use, and the information of folic acid supplementation (folic acid alone) were recorded. In the CMIHS system, a woman’s FAS information was linked to her medical records once she had her first prenatal exam in any of the local hospitals.

In this study, pregnant women who regularly took folic acid (400 μg/day) during the periconceptional period for at least 1 month were defined as “periconception users”. Of them, those who started intake before their LMP were termed as “preconception users”, while those who started on or after their LMP were named as “post-conception users”. On the contrary, pregnant women who did not have folic acid intake, or took it continuously less than 1 month were considered as “non-users”. Other variables included maternal age, nationality, education, residence, parity, medical condition and environmental exposures in the first trimester. Specifically, the maternal medical condition referred to any of such conditions as positive result of syphilis, human immunodeficiency virus and hepatitis B virus testing, anemia, chronic kidney, liver and heart diseases, diabetes (type I or II), primary hypertension disorders. The environmental exposure was an indicator (yes or no) to show whether the mothers had smoking, alcohol drinking, drug abuse, or exposure to radiation and hazardous substances. The variables of infants included gestational age, date of birth, birth weight, sex, birth defects and infant outcomes. Data used in this study were extracted anonymously, with a few variables as identifiers (e.g. date of birth). This research was approved by the Ethics Committee of West China Second University Hospital, Sichuan University. All methods were performed in accordance with relevant regulations and the individual informed consent was waived.

### Statistical analyses

Differences in maternal and infant characteristics between cases and controls were examined with Pearson chi-square tests for categorical variables. Non-conditional logistic regression analysis was used to calculate the crude and adjusted odds ratios (ORs) and 95% confidence intervals (CI). Statistical analyses were performed with R 3.5.3 (R Development Core Team 2019) and packages “rms”.

## Supplementary Information

Supplementary Information.
