# Supplementary material for: Maternal periconceptional folic acid supplementation reduced risks of non-syndromic oral clefts in offspring
Source: Sci Rep. 2021 Jun 10;11:12316. doi: 10.1038/s41598-021-91825-9 (PMC8192944; doi:10.1038/s41598-021-91825-9)
Supplement: Supplementary file 1 — Supplementary Information. [file 41598_2021_91825_MOESM1_ESM.docx]

**Maternal periconceptional folic acid supplementation reduced risks of non-syndromic oral clefts in offspring**

Wenli Xu ^1^, Ling Yi ^1^, Changfei Deng ^1^, Ziling Zhao ^2^, Longrong Ran ^3^, Zhihong Ren ^3^, Shunxia Zhao^3^, Tianjin Zhou^2^, Gang Zhang ^2^, Hanmin Liu ^4,5,6*^, Li Dai ^1, 5,6*^

^1^National Center for Birth Defects Monitoring, West China Second University Hospital, Sichuan University, Chengdu, Sichuan, China

^2^Sichuan Provincial Hospital for Women and Children, Affiliated Women and Children’s Hospital of Chengdu Medical College, Chengdu, Sichuan, China

^3^Chengdu Women’s & Children’s Central Hospital, Chengdu, Sichuan, China

^4^Pediatric Department, West China Second University Hospital, Sichuan University, Chengdu, Sichuan, China

^5^Key Laboratory of Birth Defects and Related Diseases of Women and Children (Sichuan University), Ministry of Education, Chengdu, Sichuan, China

^6^Med-X Center for Informatics, Sichuan University, Chengdu, Sichuan, China

^*^ Corresponding author:

Li Dai, National Center for Birth Defects Monitoring, West China Second University Hospital, Sichuan University, No.17 Section 3 Renminnanlu, Chengdu, Sichuan 610041, China. E-mail: [daili@scu.edu.cn;](mailto:daili@scu.edu.cn;)

Hanmin Liu, Pediatric Department, West China Second University Hospital, Sichuan University, No.17 Section 3 Renminnanlu, Chengdu, Sichuan 610041, China. E-mail: liuhm@scu.edu.cn.

Funding: This work was supported by the Grants from National Key R&D Program of China (2017YFC0907304), National Natural Foundation of China (81072375), and Ministry of Health.

Disclosure statement: The authors report no conflicts of interest. The authors alone are responsible for the content and writing of this article.

**Supplementary Table 1 The associations between selected exposures and nonsyndromic oral clefts**

| **Variables** | **NsCL/P** | **NsCL** | **NsCLP** | **NsCP** |
| --- | --- | --- | --- | --- |
|  | **(n=616)** | **(n=247)** | **(n=369)** | **(n=191)** |
| **Maternal Age (years)** |  |  |  |  |
| 25-34 | Ref | Ref | Ref | Ref |
| <25 | 0.99(0.81,1.21) | 0.98(0.71,1.34) | 1.00(0.77,1.29) | 0.85(0.59,1.21) |
| ≥35 | **1.59(1.16,2.14)** | 1.22(0.74,1.94) | **1.90(1.28,2.76)** | 1.15(0.63,1.97) |
| **Urban-rural classification** | |  |  |  |
| Urban | Ref | Ref | Ref | Ref |
| Rural | **1.26(1.04,1.54)** | 1.24(0.92,1.67) | **1.29(1.01,1.65)** | 0.96(0.70,1.32) |
| **Medical condition in the first trimester** | | |  |  |
| No | Ref | Ref | Ref | Ref |
| Yes | 0.94(0.69,1.26) | 0.92(0.55,1.43) | 0.96(0.65,1.37) | **0.46(0.20,0.89)** |
| **Environmental exposure in the first trimester** | | |  |  |
| No | Ref | Ref | Ref | Ref |
| Yes | 1.51(0.90,2.41) | 0.76(0.23,1.85) | **2.01(1.12,3.37)** | 1.59(0.55,3.62) |
| **Maternal BMI (kg/m^2^)** | |  |  |  |
| 18.5-23.9 | Ref | Ref | Ref | Ref |
| <18.5 | 0.93(0.72,1.19) | 1.12(0.76,1.61) | 0.81(0.58,1.12) | 0.77(0.48,1.19) |
| ≥24 | **1.27(1.03,1.56)** | **1.55(1.13,2.09)** | 1.10(0.84,1.44) | 1.11(0.76,1.59) |
| **Parity** |  |  |  |  |
| Nulliparous | Ref | Ref | Ref | Ref |
| Multiparous | **0.79(0.64,0.97)** | 1.00(0.73,1.37) | **0.67(0.51,0.87)** | 0.93(0.65,1.30) |
| **Infant sex** |  |  |  |  |
| Female | Ref | Ref | Ref | Ref |
| Male | **1.48(1.25,1.75)** | **1.49(1.15,1.94)** | **1.46(1.18,1.81)** | **0.71(0.53,0.95)** |

NsCL/P, nonsyndromic cleft lip with or without cleft palate cases; NsCL, nonsyndromic cleft lip cases; NsCLP, nonsyndromic cleft lip with cleft palate cases; NsCP, nonsyndromic cleft palate cases.
